# A Removable 3D-Printed Protective Cap for Chronic Cranial Implants in Rhesus Macaques (*Macaca mulatta*): A Descriptive Case Series and Technical Refinement

**DOI:** 10.3390/ani16182828

**Published:** 2026-09-08

**Authors:** Rochelle D. Moore, Dorsa Amirkhany, Kelsey Clark, Behrad Noudoost

**Affiliations:** Department of Ophthalmology and Visual Sciences, Moran Eye Center, University of Utah, Salt Lake City, UT 84132, USA; dorsa.amirkhany@gmail.com (D.A.); behrad.noudoost@utah.edu (B.N.)

**Keywords:** cranial implant protection, 3D-printed cap, wound healing, ophthalmology, neuroscience, visual science, rhesus macaques, neural recordings, neurological disorders, neurophysiology, behavioral studies

## Abstract

Chronic neurophysiology studies in Rhesus macaques (*Macaca mulatta*) require implantation of cranial hardware including headposts, recording chambers, and fixation screws. A common complication is persistent picking, scratching and grooming around implant margins, which can result in skin retraction, chronic inflammation, infection, repeated surgical repairs, and premature removal of the animal from research studies. Existing implant-care protocols rely heavily on topical therapies that may inadvertently increase grooming behavior. We developed a removable carbon fiber 3D-printed cranial cap that attaches directly to the headpost, covering the implant margins while allowing airflow and rapid removal during recording sessions. Three macaques received the protective 3D-printed cap. One animal tolerated the device for 836 days, during which implant manipulation ceased, surgical repair frequency decreased, and an additional 1056 h of electrophysiological data were collected. A second animal successfully used the 3D-printed cap during postoperative healing for 61 days following implant revision surgery. One animal did not tolerate the 3D-printed cap, and it was removed. This case study suggests that a simple, low-cost 3D-printed cap has the potential to improve wound healing, reduce implant manipulation, enhance animal welfare, and prolong the functional lifespan of chronic cranial implants in appropriately selected animals.

## Simple Summary

Brain research in Rhesus macaques often requires surgically implanted devices, such as headposts and recording chambers, to measure brain activity over long periods. Monkeys naturally scratch and groom around these implants, which can damage the skin, delay healing, increase the risk of infection, and sometimes require additional surgeries or early removal from research studies. Current treatments, such as topical wound products, may unintentionally encourage more scratching. To address this problem, we developed a lightweight, removable 3D-printed carbon fiber cap that fits over the implants and acts as a protective barrier. The 3D-printed cap attaches securely with two screws, is easy to remove before research sessions, and can be sterilized in an autoclave. The 3D-printed caps were tested in three *Rhesus macaques*. Two out of three animals tolerated the 3D-printed caps. After introducing the cap, the frequency of surgical repairs was reduced for one animal that previously required multiple interventions. These caps have the potential to reduce complications, improve healing, decrease the need for repair surgeries, and support better animal welfare while allowing researchers to collect scientific data.

## 1. Introduction

Rhesus macaques are central to modern science, especially neuroscience, because their brains, behavior and genetics closely parallel our own, making them one of the most translational models for understanding the human mind. The rhesus genome is about 93% similar to the human genome, and their brain architecture follows the same organizational principles as ours, with many homologous cortical areas. This similarity makes them ideal for studying memory, decision making, social cognition and neurological diseases. Rhesus macaques have been indispensable for decades because their cognitive abilities are sophisticated—they excel at tasks involving working memory [1], attention and executive function, allowing researchers to study high-level brain processes. Their neural systems mirror human systems, enabling investigation of disorders such as Alzheimer’s disease [2], age-related cognitive decline, and other neurodegenerative conditions. Their social behavior is rich and complex, making them well-suited for studying the neural basis of social interaction, empathy and conflict. One recent study recorded neural activity from freely interacting macaques, revealing how their prefrontal cortices encode social behaviors like grooming, reciprocity, and responses to aggression [3]. This kind of naturalistic neuroscience is nearly impossible in other species. Even with advances in AI, organoids and computational modeling, Rhesus macaques remain essential because they allow single-neuron recordings during complex behaviors, they exhibit natural social dynamics that can be studied at the neural level, and they provide a living, behaving system that mirrors human cognition in ways no other model can.

Despite these scientific benefits, chronic cranial implants present ongoing veterinary challenges [4,5]. Common complications include:

1. Skin margin retraction between the headpost and cylinder chambers.
2. Chronic inflammation.
3. Bacterial infection.
4. Bone degradation (osteomyelitis).
5. Implant instability.

Approximately 30% of macaques with chronic cranial implants experience at least one re-suturing surgery, and many require repeated veterinary intervention [6]. Routine wound care, including antiseptics, enzymatic debridement, and topical medications, is effective but often stimulates additional picking, scratching and grooming around the surgical margins. In our experience, this risks creating a cycle of repeated tissue trauma, delayed healing, and increased infection risk.

Protective head coverings have previously been described as reducing postoperative complications [6]. However, our laboratory required a device that could be:

1. Rapidly removed before each recording session.
2. Sterilized by autoclaving.
3. Compatible with existing headposts and recording chambers.
4. Durable enough for long-term use.

## 2. Case Descriptions

### 2.1. Overview

**Figure 1:**
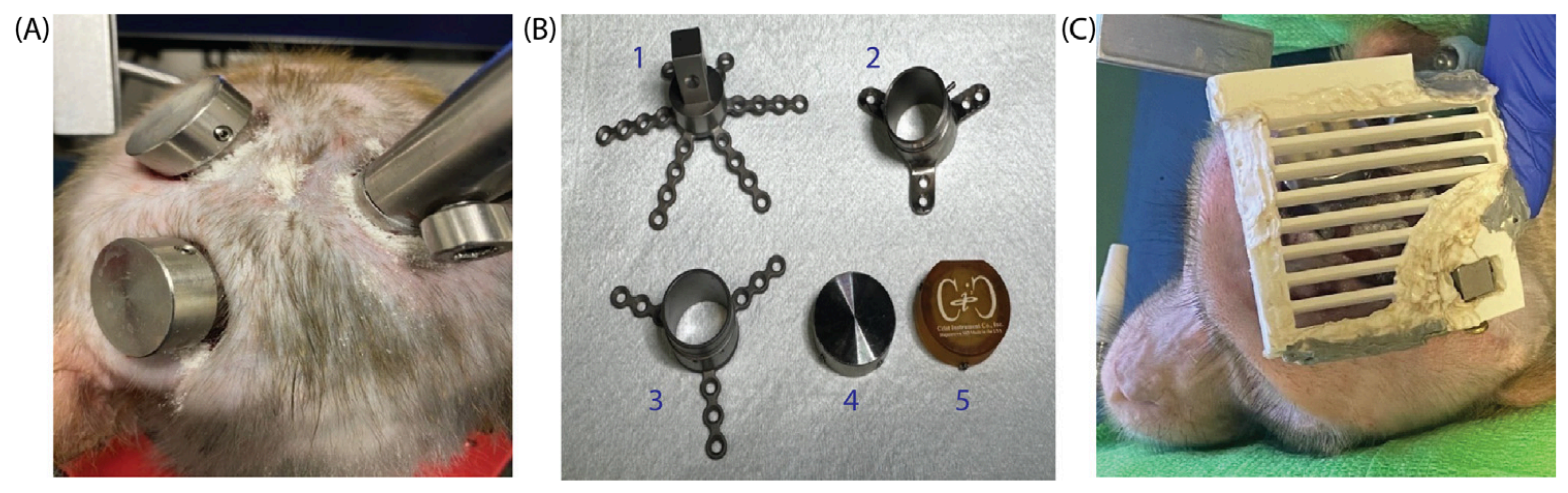
Cranial hardware and prototype PLA cap. (**A**) Example of healed implant. Animal T healed with two cylindrical recording chambers and head fixed with headpost, following removal of 3D-printed cap. Vetrix Healion Amniotic Matrix powder (Vetrix, Dawsonville, GA, USA) was applied around hardware (Gray Matter titanium recording chambers with custom stainless cap; Gray Matter Research, Bozeman, MT, USA). (**B**) Cranial hardware: 1. Gray Matter titanium headpost; 2. Crist Instruments stainless steel recording chamber (Crist Instruments Co., Inc., Haggerstown, MD, USA); 3. Gray Matter titanium recording chamber; 4. Custom stainless steel cap; 5. Crist Instruments cap. (**C**) Early prototype consisting of 3D-printed grids made with PLA filament and soldered together.

Here we provide a qualitative description of our experiences using a 3D-printed cap on three male rhesus macaque monkeys (4–12 years old) participating in chronic neurophysiology studies at the University of Utah. Our design and introduction of the 3D-printed cap were motivated by our experiences over decades of research, in which animals were sometimes removed from study due to health complications arising from persistent manual manipulation of implant margins. In summary, one animal (animal E) wore a 3D-printed cap for an extended period, which may have contributed to improved wound management and well-being; one animal (animal T) wore a 3D-printed cap for two months during surgical recovery (Figure 1A); and one animal (animal O) did not tolerate the 3D-printed cap. These cases are discussed in greater detail below.

### 2.2. Methods

*General surgical procedures.* All surgical procedures and neurophysiological experiments were performed under protocols approved by the Institutional Animal Care and Use Committee and conducted according to NIH Guidelines for the Care and Use of Laboratory Animals. Prior to introducing the 3D-printed cap, all animals had undergone multiple stages of surgical implantation, including titanium headpost placement, osseointegration, recording chamber implantation, and chamber craniotomies. Cranial hardware is shown in Figure 1B. Routine postoperative care included analgesia, antimicrobial prophylaxis, wound cleaning, and regular veterinary monitoring.

*Monitoring and enrichment.* Animals were monitored 2–3 times daily by veterinarians, veterinary technicians, husbandry staff, and lab personnel. We monitored the animals’ food and water intake, stool and urine output, any signs of pain (hunched posture, lethargy and teeth grinding), play with toys, foraging for treats, and ability to move freely in the home cage. ‘Manipulation’ describes the animal picking, scratching, or otherwise manually interacting with the wound margin adjacent to cranial hardware, or the hardware itself. We rotated toys daily from vendors such as Bio-serv (Bio-Serv, Flemington NJ USA) and Otto Environmental (Otto Environmental, Greenfield WI USA), with hand-held mirrors always available. Destructible enrichment was also given several times a week. Enrichment is a daily requirement for non-human primates [7]. For 30 days following initial placement of the 3D-printed cap, we increased enrichment by 1.5–2x/day to reduce novelty-related manipulation. After first attaching the cap, we initially observed the animal every couple of hours during a 6 am–6 pm circadian light cycle for the first week, then 2–3 times a day for 2–3 more weeks, then returned to standard daily monitoring. At the request of the veterinary team, we chaired the animal and removed the 3D-printed cap for evaluation of wound healing.

*3D-printed cap design.* We initially designed a polylactic acid (PLA) prototype (Figure 1C). Here we focus on the revised design, a removable 3D-printed cap using a carbon fiber-reinforced PA12 filament, printed on a Raise3D E2CF printer (Raise3D, Stafford, TX, USA).

**Figure 2:**
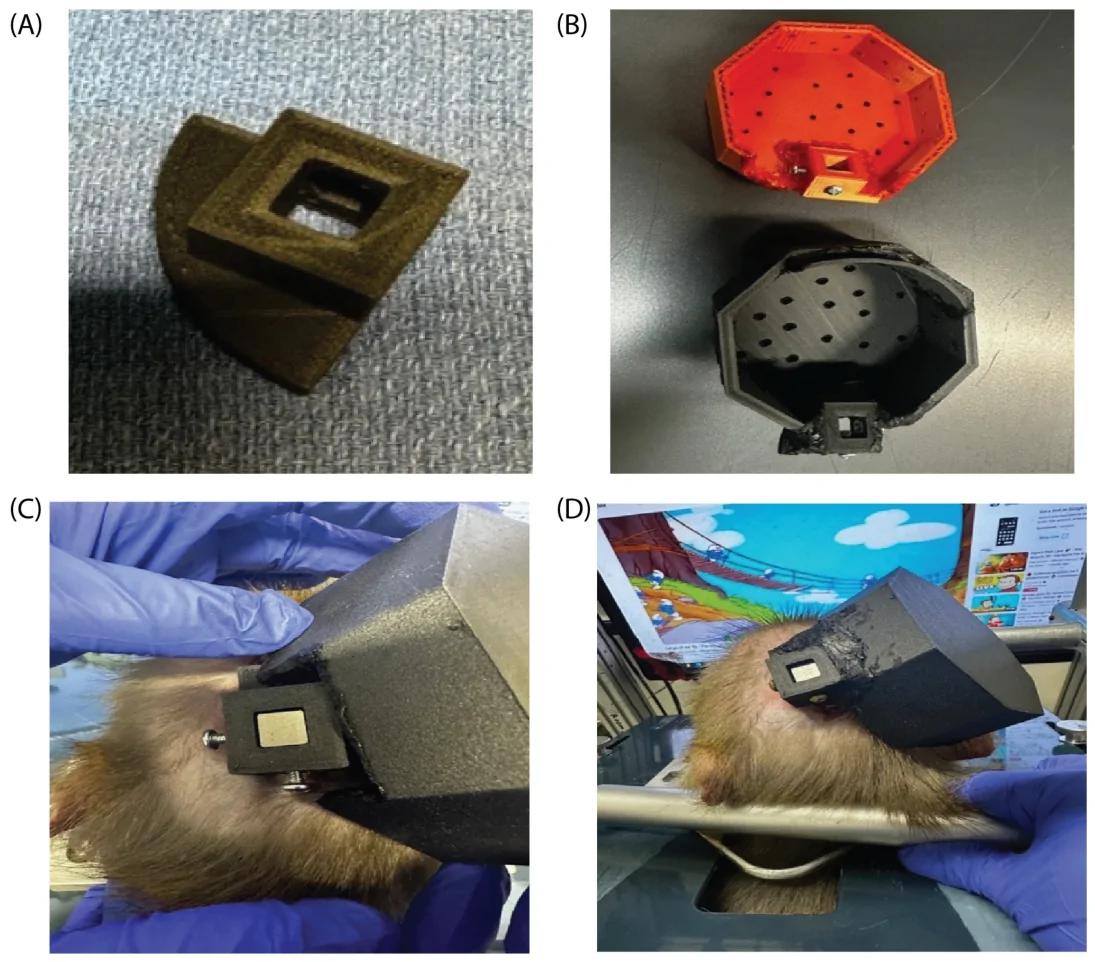
3D-printed carbon fiber cap. (**A**) 3D-printed carbon fiber headpost connector. (**B**) Orange PLA printed cap and black carbon fiber 3D-printed caps, soldered and ready for use. (**C**) Animal E being fitted with headpost connector and cap prior to soldering them together. (**D**) Animal E with 3D-printed carbon fiber cap soldered to headpost piece, covering all cranial hardware.

The cap consisted of two components:

1. A custom headpost connector was secured with two screws and washers (Figure 2A). (Design file provided as Supplemental Material). The connector piece was designed separately and soldered to the octagonal shape, as each animal’s orientation of the implants was slightly different, and on different hemispheres of the skull.
2. An octagonal protective shell covers both recording chambers and the surrounding skin margins (Figure 2B; open source STL file, ‘N-sided boxes’, https://www.thingiverse.com/thing:27716, accessed on 21 May 2024).

Relative positioning and orientation of the headpost connector and the octagonal shell were adjusted and marked by hand while an awake animal sat in the primate chair (Figure 2C), then the two pieces were soldered together on a workbench. The assembled cap attached to the headpost is shown in Figure 2D. Multiple ventilation holes were incorporated into the cap using a high-heat soldering iron to promote airflow while preventing access by the animal’s fingers. This also prevented the accumulation of moisture and debris under the cap. The cap required approximately 6 h 27 min to print, and $13.20 in materials per unit.

Unlike earlier PLA prototypes that fractured under repeated manipulation, the carbon fiber version was substantially stronger, sterilizable using a steam autoclave, and more easily removable before recording sessions. We autoclaved our caps twice weekly (250F for 15 min), or as needed, without signs of deterioration over 2–3 months of use.

*Experimental workflow.* For animal E, who wore the 3D-printed cap for an extended period while participating in neurophysiological recordings, the cap was removed at the beginning of and replaced at the end of each recording session, while the animal sat in the primate chair with head unrestrained. It did not interrupt recording sessions, as it took only a few seconds to unscrew and remove prior to recording, and place back on and tighten two screws after recording.

### 2.3. Case 1: Long-Term Use

In March of 2022, we designed our pilot cap using 3D-printed PLA grids, for animal E. Animal E was implanted in January 2021, and had developed some skin retraction (approximately 1” in diameter). Animal E consistently picked at his implant and margin areas on a daily basis, resulting in skin retraction and ultimately requiring multiple revision surgeries (four over 6 years prior to the cap placement). Attempts to discourage this behavior by providing an abundance of enrichment and various forms of entertainment (television, destructible items, toys, food, etc.) were not successful. Revision surgeries were required when more than one of the multiple titanium screws securing one of the legs of an implant were exposed from the skin after retraction. We also used Low Level Laser Therapy around the implants—183 treatments (1–250 Hz; 5 min sessions over 24 months) with no significant improvement [8].

Animal E was fitted with a 3D-printed cap, which physically prevented him from picking at the skin underneath. He wore the cap (first the PLA prototypes, and subsequently the 3D-printed carbon fiber cap) for 836 days while participating in neurophysiological experiments, with no need for additional revision surgeries in that time. By preventing physical access to the cranial hardware and wound margin, the cap should also allow topical therapies to stay in place longer. Our observation was that following cap placement, wound healing improved, no systemic antibiotics were required, and surgical repair frequency decreased (no repair surgeries over 836 days following cap placement, compared to four repair surgeries over 2242 days prior to introducing the cap).

Our research team recorded 1263 h over 2242 days prior to placing the 3D-printed cap, and an additional ~1056 h of electrophysiological recordings over 836 days following introduction of the cap. We cannot definitively state that the 3D-printed cap was necessary for these additional recordings, but given the animal’s history of repair surgeries it is our opinion that without the cap there would likely have been additional surgical repairs and loss of recording time, or even removal from the study altogether. After experiencing success introducing the 3D-printed cap in Animal E, we subsequently tried introducing a cap with the other two animals in the lab (cases 2 & 3, described below).

### 2.4. Case 2: Postoperative Protection

Animal T was fitted with a 3D-printed cap after a major revision surgery, where we moved one of his existing recording chambers to another location. He wore his 3D-printed cap for 61 days, which allowed ample healing time. Once the 3D-printed cap was removed, he did not pick at the implant area, and the cap was permanently removed. This animal has been in the lab for 3017 days and has worked 1209 h as of August 2026.

### 2.5. Case 3: Device Intolerance

Animal O was fitted with a 3D-printed cap after a revision surgery, but was forceful and persistent with his manual manipulation of the cap immediately following its introduction. Within six hours of initial placement, the cap broke at the soldering point (where the connector piece joins the chamber). Although it is possible that with a more extensive and gradual introduction the cap might have been tolerated, in consultation with the veterinary staff it was decided that cap use should be discontinued in this animal.

Despite not having a cap, animal O has been in the lab for 3129 days and has worked 5239 h as of August 2026, with one surgical repair since the failed attempt to introduce the cap. Our ability to record from this animal without a 3D-printed cap is consistent with the fact that similar neurophysiological research has been conducted in many labs for decades without using such caps. This outcome highlights the importance of individual behavioral differences in determining an animal’s suitability for this refinement.

### 2.6. Summary

Among animals that tolerated the 3D-printed cap, investigators observed fewer implant-margin manipulations, improved wound healing, fewer repair surgeries (for animal E, four prior to and none after placing the 3D-printed cap), reduced need for antibiotic treatment, and increased availability for recording sessions (compared to prior to introduction of the cap). The veterinary staff supports attempting to use these 3D-printed caps in the future for all animals who undergo surgical implant or implant repair procedures.

The limited sample size, lack of control group, and descriptive nature of this case series do not allow us to definitively attribute these outcomes to the 3D-printed cap; however, other researchers may wish to consider this option for animals experiencing negative complications due to manipulation of the implant or skin margin.

## 3. Discussion

This case study demonstrates the potential value of a removable 3D-printed cap as a refinement for chronic cranial implant management in *Rhesus macaques*. The 3D-printed cap functions primarily as a physical barrier that interrupts the cycle of picking, scratching, tissue trauma, inflammation, and infection that frequently complicates long-term cranial implants. By preventing manual access to implant margins while allowing ventilation, the device may promote healing without interfering with normal implant maintenance.

Previously published work describes moldable protective caps that cover the entire head, which have been used during surgical healing [6]. One difference between our design and this prior publication is that their cap is formed from moldable material while the animal is anesthetized, while ours is 3D-printed and then customized with an awake animal in a primate chair. It is not clear whether this previously published design can be quickly and easily removed and replaced during the workflow of a standard experimental session, as ours can. On the other hand, their molded design is lower profile, which may reduce the chance of breakage, and may provide better ventilation. Overall this previous publication provides a larger sample size and quantitative validation of the same principle motivating our own use of caps: that a physical barrier can aid in healing following surgical implants.

Importantly, the 3D-printed cap is not appropriate for every animal. Behavioral tolerance should be assessed individually, and removal should occur promptly if the device itself becomes a source of distress or excessive manipulation.

### 3.1. Animal Welfare Implications

This refinement directly supports the Refinement principle of the Three Rs [9] by reducing discomfort associated with chronic cranial implants without altering experimental objectives. Potential benefits include reduced self-trauma, improved surgical healing, fewer repeat surgical procedures, reduced infection risk, decreased antibiotic use, prolonged implant stability, and increased time available for scientific data collection.

By potentially extending implant longevity while improving health outcomes, the 3D-printed cap may also indirectly contribute to a reduction in the number of animals used by maximizing data obtained from individual animals and decreasing the need for replacement subjects.

### 3.2. Limitations

This report is intended to share our descriptive observations on the efficacy of a relatively simple potential means of mitigating what is, in our experience, a frequent challenge in primate neurophysiological research. The descriptive case series here includes only three animals, with no formal comparison group or randomization, and no quantitative behavioral or wound healing scoring system. We were able to use a single printed carbon-fiber cap for over two months with twice-weekly autoclaving; establishing the limits and average long-term durability of the cap with repeated autoclaving and mechanical stress will require further data. The cap system does require the animal to be removed from their cage in order to remove the cap and examine the wound. Adaptation of the design would be required to attach it to different hardware systems, or to accommodate animals with a different spatial arrangement of cranial hardware. As noted, not all animals tolerated the cap, requiring careful and frequent behavioral observation when the cap is first introduced.

## 4. Conclusions

This case study describes the implementation of a removable 3D-printed cap for Rhesus macaques with chronic cranial implants. In animals that tolerated the 3D-printed cap, use coincided with reduced implant manipulation, improved wound management, and decreased surgical interventions over extended periods of study participation. While larger studies are needed to establish and quantify efficacy, this inexpensive and customizable refinement represents a promising addition to chronic cranial implant management and may support improved animal welfare in long-term neuroscience research.

## Data Availability

No new data were created or analyzed in this study. Data sharing is not applicable to this article.

## Supplementary Materials

The following supporting information can be downloaded at: https://www.mdpi.com/article/10.3390/ani16182828/s1. Supplementary File S1: Digital design file for the headpost connector.
